# Assessment of Posttraumatic Stress Disorder and Educational Achievement in Sweden

**DOI:** 10.1001/jamanetworkopen.2020.28477

**Published:** 2020-12-08

**Authors:** Alba Vilaplana-Pérez, Anna Sidorchuk, Ana Pérez-Vigil, Gustaf Brander, Kayoko Isoumura, Eva Hesselmark, Laura Sevilla-Cermeño, Unnur A. Valdimarsdóttir, Huan Song, Andreas Jangmo, Ralf Kuja-Halkola, Brian M. D’Onofrio, Henrik Larsson, Gemma Garcia-Soriano, David Mataix-Cols, Lorena Fernández de la Cruz

**Affiliations:** 1 Centre for Psychiatry Research, Department of Clinical Neuroscience, Karolinska Institutet, Stockholm, Sweden; 2 Stockholm Health Care Services, Region Stockholm, Stockholm, Sweden; 3 Departament de Personalitat, Avaluació i Tractaments Psicològics, Universitat de València, València, Spain; 4 Department of Child and Adolescent Psychiatry and Psychology, Institute of Neuroscience, Hospital Clínic de Barcelona, Barcelona, Spain; 5 Science for Life Laboratory, Department of Medical Biochemistry and Microbiology, Uppsala University, Uppsala, Sweden; 6 Departamento de Medicina y Especialidades Médicas, Universidad de Alcalá, Madrid, Spain; 7 Department of Medical Epidemiology and Biostatistics, Karolinska Institutet, Stockholm, Sweden; 8 Center of Public Health Sciences, Faculty of Medicine, University of Iceland, Reykjavík, Iceland; 9 Department of Epidemiology, Harvard T.H. Chan School of Public Health, Boston, Massachusetts; 10 Biomedical Big Data Center, West China Hospital, Sichuan University, Chengdu, Sichuan, China; 11 Department of Psychological and Brain Sciences, Indiana University, Bloomington; 12 School of Medical Sciences, Örebro University, Örebro, Sweden

## Abstract

**Importance:**

Posttraumatic stress disorder (PTSD) has been associated with impaired educational performance. Previous studies on the disorder could not control for important measured and unmeasured confounders.

**Objective:**

To prospectively investigate the association between PTSD and objective indicators of educational attainment across the life span, controlling for familial factors shared by full siblings, psychiatric comorbidity, and general cognitive ability.

**Design, Setting, and Participants:**

This population-based cohort study included 2 244 193 individuals born in Sweden between January 1, 1973, and December 31, 1997, who were followed-up until December 31, 2013. Clusters of full siblings were used to account for familial factors. Data analyses were conducted between December 2018 and May 2020.

**Exposure:**

*International Classification of Diseases*, *Ninth Revision* and *International Statistical Classification of Diseases and Related Health Problems*, *Tenth Revision* diagnoses of PTSD in the Swedish National Patient Register.

**Main Outcomes and Measures:**

Eligibility to access upper secondary education after finishing compulsory education, finishing upper secondary education, starting a university degree, and finishing a university degree.

**Results:**

Of the final cohort of 2 244 193 individuals (1 151 414 [51.3%] men) included in the analysis, 1 425 326 were assessed for finishing compulsory education (919 with PTSD), 2 001 944 for finishing upper secondary education (2013 with PTSD), and 1 796 407 and 1 356 741 for starting and finishing a university degree (2243 and 2254 with PTSD, respectively). Posttraumatic stress disorder was associated with lower odds of achieving each of the educational milestones during the study period, including 82% lower odds of finishing compulsory education (adjusted odds ratio [aOR], 0.18; 95% CI, 0.15-0.20), 87% lower odds of finishing upper secondary education (aOR, 0.13; 95% CI, 0.12-0.14), 68% lower odds of starting a university degree (aOR, 0.32; 95% CI, 0.28-0.35), and 73% lower odds of finishing a university degree (aOR, 0.27; 95% CI, 0.23-0.31). Estimates in the sibling comparison were attenuated (aOR range, 0.22-0.53) but remained statistically significant. Overall, excluding psychiatric comorbidities and adjusting for the successful completion of the previous milestone and general cognitive ability did not statistically significantly alter the magnitude of the associations.

**Conclusions and Relevance:**

Posttraumatic stress disorder was associated with educational impairment across the life span, and the associations were not entirely explained by shared familial factors, psychiatric comorbidity, or general cognitive ability. This finding highlights the importance of implementing early trauma-informed interventions in schools and universities to minimize the long-term socioeconomic consequences of academic failure in individuals with PTSD.

## Key Points

**Question:**

To what extent is posttraumatic stress disorder (PTSD) associated with impaired educational performance over and above familial factors, psychiatric comorbidity, and general cognitive ability?

**Findings:**

This population-based cohort study of 2 244 193 individuals found that those diagnosed as having PTSD were statistically significantly less likely to achieve all educational milestones, from compulsory education to finishing university, compared with individuals without the disorder independent of familial factors shared between siblings, psychiatric comorbidity, and general cognitive ability.

**Meaning:**

Findings of this study suggest that posttraumatic stress disorder is associated with lower educational performance across the life span and independent of familial factors, psychiatric comorbidity, and general cognitive ability.

## Teaser

This population-based cohort study investigates the association between posttraumatic stress disorder and objective indicators of educational attainment across the life span, controlling for familial factors shared by full siblings, psychiatric comorbidity, and general cognitive ability.

## Introduction

Posttraumatic stress disorder (PTSD) is a common psychiatric condition, with a lifetime prevalence of 5.6% among individuals exposed to trauma and 3.9% in the general population.^1^ Known risk factors for PTSD include genetic factors, female sex, preceding somatic diseases, family history of psychiatric disorders, cumulative exposure to traumatic experiences, higher severity of the traumatic events, and low premorbid cognitive ability.^2,3,4,5^ Individuals with PTSD have high rates of psychiatric comorbidity,^6^ multiple adverse health consequences,^7,8,9^ and high rates of suicide.^10,11^ Posttraumatic stress disorder is associated with substantial functional impairment, including problems in relationships and family functioning^12,13^ and work-related disabilities.^14^ The deleterious implications of PTSD for educational performance have been suggested in a small number of primarily cross-sectional studies.^15,16,17,18,19^ Longitudinal studies have been even scarcer but also suggest that PTSD can impair educational performance.^17,19^

Although informative, these previous studies^15,16,17,18,19^ had several limitations, including cross-sectional designs, focus on a single educational milestone, generally small samples, self-reported educational achievements, or insufficient control of important confounders (eg, familial factors, psychiatric comorbidity, and general cognitive ability). Accounting for such potential confounders is essential because the association between PTSD and education is likely to be complex for several reasons. First, because genome-wide association studies^4,5,20,21^ indicate that both PTSD vulnerability and educational achievement have a genetic component, it is possible that pleiotropic genetic effects may be at play, whereby a shared familial or genetic vulnerability may explain both the increased risk of PTSD and the poor scholastic attainment in trauma-exposed individuals. Second, PTSD is frequently comorbid with psychiatric disorders, which are in turn known to impair educational achievement.^22,23,24,25^ Third, PTSD has been associated with low premorbid cognitive ability,^2,26^ which correlates with educational attainment.^27,28^ Given the combined association of these potential confounders, it is unclear to what extent PTSD per se disrupts education.

We aimed to investigate the association between PTSD and objective indicators of educational attainment across the life span using the Swedish national registers, which include independently and prospectively collected health care and academic data from primary to tertiary education for the whole population. To reduce the impact of possible confounders on this association, we conducted a sibling comparison, systematically evaluated the role of psychiatric comorbidities, and adjusted for a number of relevant variables.

## Methods

The Stockholm Regional Ethical Review Board approved this population-based cohort study. Because all individuals included in our register-based study were deidentified, the requirement for informed consent was waived by the review board. This study followed the Strengthening the Reporting of Observational Studies in Epidemiology (STROBE) reporting guideline.^29^

### Data Sources and Design

We linked various Swedish nationwide registers through the unique personal identification number assigned to all residents at birth or immigration.^30^ We used the Total Population Register^31^ and the Cause of Death Register^32^ to identify included and excluded individuals. In addition, we retrieved information from the National Patient Register (NPR),^33^ which uses codes from the *International Classification of Diseases* (*ICD*) and the Multi-generation Register,^34^ that links individuals to their parents and allows identification of relatives. Information on education was gathered from the National School Register,^35^ which contains information on educational attainment from all schools, and the Longitudinal Integrated Database for Health Insurance and Labour Market Studies (Swedish acronym LISA),^36^ which provides annual data on education, labor market, and social sectors. We also included information from the Conscription Register,^37^ which contains information about the health examination of individuals at military conscription between 1969 and 2010.

### Study Population

**Figure 1.**
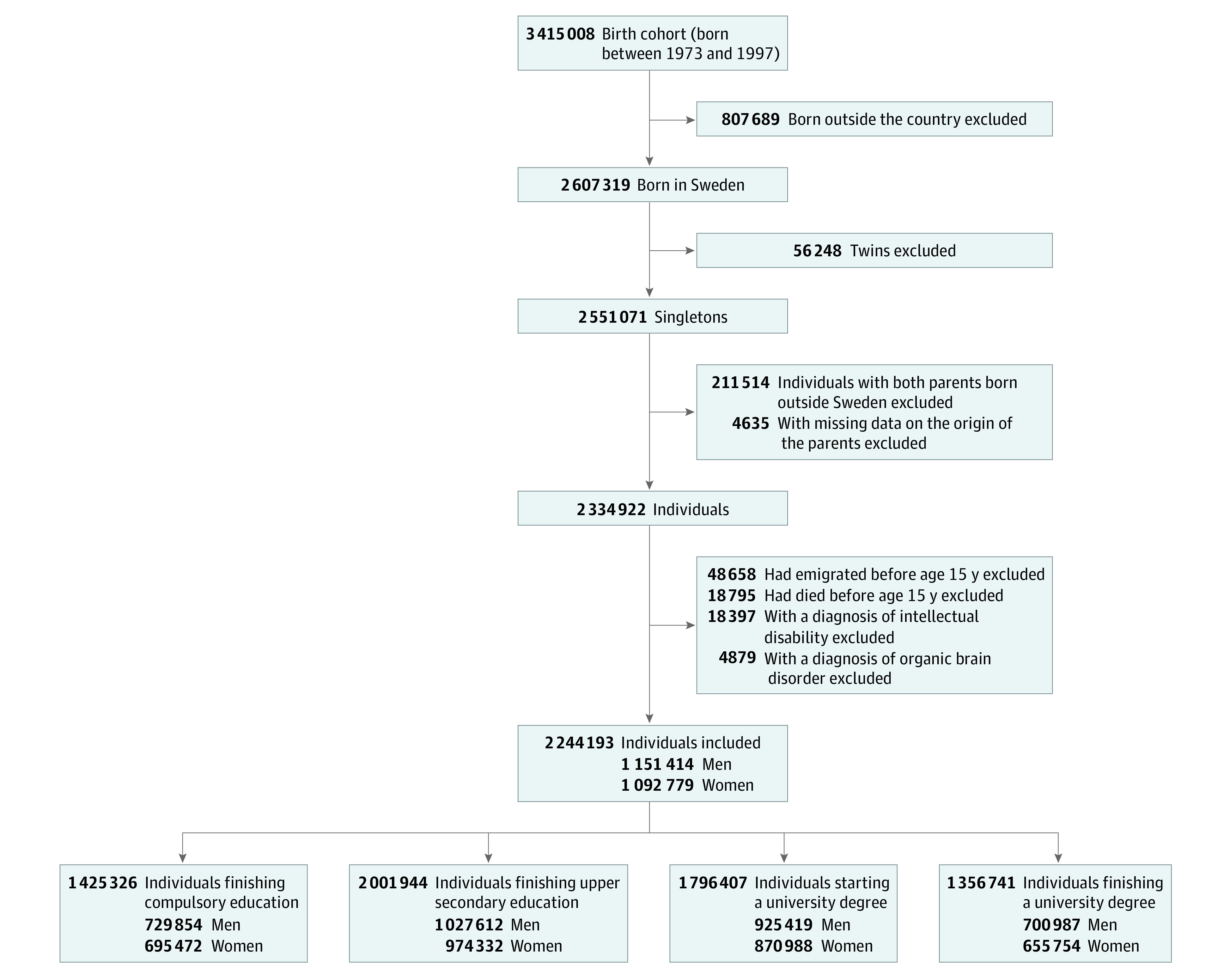
Flowchart of the Study Population The final study cohort comprised 2 244 193 individuals (51.3% male and 48.7% female).

The initial cohort consisted of all singleton births in Sweden between January 1, 1973, and December 31, 1997, totaling 2 551 071 individuals (1 306 149 men [51.2%] and 1 244 922 women [48.8%]). Because second-generation immigrants have been reported to have lower educational performance owing to language barriers (ie, their first language is different from the language spoken in the host community),^38^ we excluded individuals with 2 parents born outside Sweden (or with missing data on parental origin) to control by restriction for this potential confounder. We also excluded individuals who had emigrated or died before age 15 years (the expected minimal age of graduation from compulsory education in Sweden) and individuals diagnosed as having intellectual disabilities or organic brain disorders (Figure 1). eTable 1 in the Supplement lists *ICD* codes and minimal age thresholds for diagnosis of psychiatric disorders. The final study cohort of 2 244 193 individuals was followed up until December 31, 2013, for their educational attainment.

**Figure 2.**
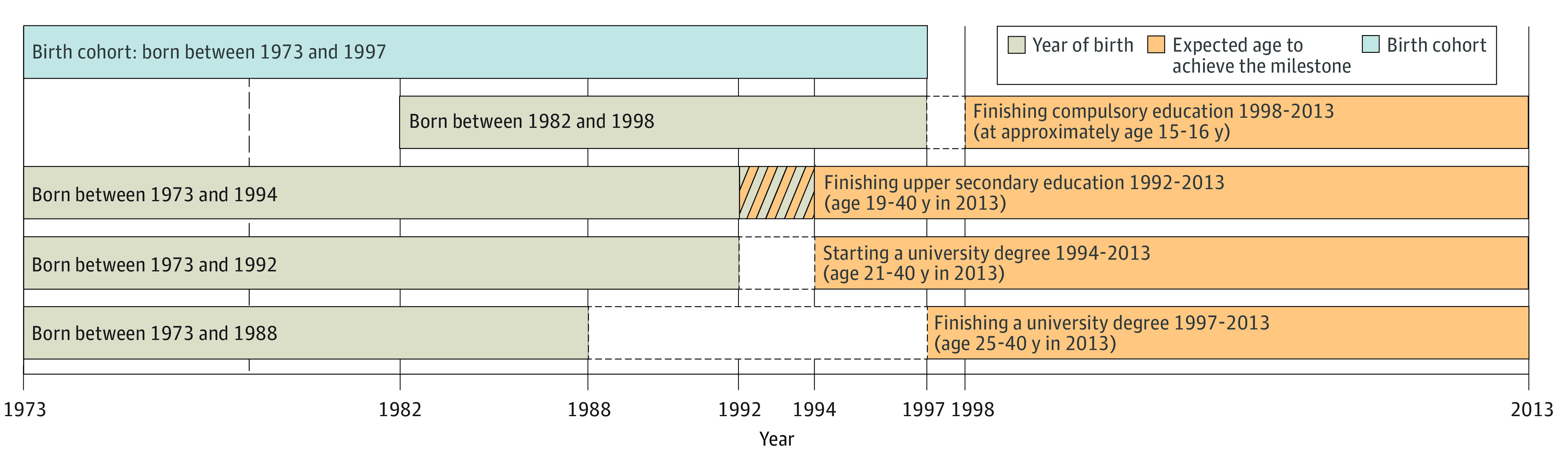
Distribution of the Study Population Shown is selection of subcohorts from the main birth cohort according to each educational milestone. The diagonal lines indicate the years when some individuals of the cohort were born and overlap with the oldest individuals of the cohort being old enough to finish upper secondary education. The white boxes in the middle of the figure framed by dashed lines indicate the years between when the individuals of the cohort were born and when they were not old enough to achieve the milestone.

Next, we defined subcohorts (Figure 2) to explore the association between PTSD and the educational milestones under study. Each subcohort comprised only individuals who had sufficient time to achieve every separate educational level studied and who did not emigrate or die before the expected age of achievement of each milestone (according to Statistics Sweden^39^). For the sibling comparison, we identified a subsample of families within each subcohort that comprised at least 2 singleton full siblings (ie, those sharing both parents) discordant for the diagnosis of PTSD. To adjust for general cognitive ability, we selected a subsample of men from the Conscription Register within each subcohort born in Sweden between 1973 and 1993 who were assessed for this measure at approximately age 18 years in the context of the conscription testing.

### Exposure

Individuals diagnosed as having PTSD according to the *International Classification of Diseases*, *Ninth Revision* (*ICD-9*) (code 309B) and according to the *International Statistical Classification of Diseases and Related Health Problems*, *Tenth Revision* (*ICD-10*) (code F43.1), as recorded in the NPR (with nationwide coverage for inpatient psychiatric visits from 1973 and outpatient psychiatric visits from 2001), were considered exposed. The Swedish *ICD* codes for PTSD are valid and reliable.^40^ To capture the association between PTSD and educational achievement within each subcohort, we collected the diagnosis of PTSD recorded at age 6 years or older (to avoid misclassifications) but before the expected age of completing each educational milestone. Therefore, for each subcohort, PTSD diagnoses were used to denote the exposure status if recorded between age 6 and 16 years for finishing compulsory education, between age 6 and 19 years for finishing upper secondary education, between age 6 and 21 years for starting a university degree, and between age 6 and 25 years for finishing a university degree. For each educational milestone, individuals with no PTSD diagnoses recorded in the corresponding age interval were considered unexposed.

### Outcomes

#### Compulsory Education

The Swedish primary education and lower secondary education are compulsory and take 9 years to complete (generally finished at age 15-16 years). Because of the changes introduced in 1998 in the grading system and thus in the eligibility criteria to access upper secondary education, we retrieved information from the National School Register only for the subcohort of individuals graduating between 1998 and 2013 (n = 1 425 326), who had comparable eligibility scores. Individuals were eligible to access upper secondary education (vs not eligible) if they attained a passing grade in 3 core subjects, including Swedish, English, and mathematics (and since 2011 in 5 additional subjects^41^).

#### Educational Attainment After Compulsory Education

From the LISA database, we retrieved information about individual data on achieving (vs not achieving) 3 post–compulsory education levels among the members of the corresponding subcohorts. The 3 levels were finishing upper secondary education (n = 2 001 944), starting a university degree (n = 1 796 407), and finishing a university degree (n = 1 356 741).

### Covariates

From the Total Population Register, we collected information on sex, birth year, and parental age at childbirth. From the NPR, we extracted lifetime information for each study participant on the following psychiatric disorders: (1) neurodevelopmental disorders (including autism spectrum disorder, attention-deficit/hyperactivity disorder, Tourette syndrome and chronic tic disorder, and learning disabilities); (2) conduct disorder; (3) phobic, anxiety, and obsessive-compulsive disorders; (4) affective disorders (including bipolar, depressive, and persistent mood disorders); (5) eating disorders; (6) psychotic disorders (including schizophrenia, schizotypal, and delusional disorders); and (7) substance use disorders (eTable 1 in the Supplement lists *ICD* codes and age thresholds). A combined “any psychiatric comorbidity” variable was also created. From the Conscription Register, we retrieved information on general cognitive ability. This measure was assessed by means of the Swedish Enlistment Battery,^42^ which included subtests that measured logical, spatial, verbal, and technical abilities, generating a stanine (9-point) categorical score (mean [SD], 5 [2] points), with higher scores indicating greater abilities.

### Statistical Analysis

Data analyses were conducted between December 2018 and May 2020. First, the association between PTSD and each educational outcome was assessed with logistic regression models to obtain odds ratios (ORs) and corresponding 95% CIs. Each outcome was assessed within the corresponding subcohort (ie, among the individuals who were alive and living in Sweden at the age old enough to start or complete a corresponding educational level). Crude models were followed by models adjusted for sex (binary variable), birth year (continuous variable), and maternal and paternal age at childbirth (categorized as a 5-year increment). To account for dependence between repeated observations within families, all models were clustered by the mother’s identification number and a robust sandwich estimator of SEs.^43^ Despite that the Swedish educational system is not linear and there are several ways of reentering the system, we conducted an additional analysis in which the models were adjusted for the outcome completed in the previous milestone (achieved vs not achieved) in an attempt to control for a potential carryover association of not passing a previous milestone.

Second, a conditional logistic regression model was used for the sibling comparison analysis within the subsample of full siblings discordant for PTSD, conditional on family identification number. By design, the model controls for familial confounders shared by full siblings (ie, about 50% of the genetic load and shared environmental factors, including socioeconomic status and stable parental traits). Within a family, unexposed siblings served as controls to the exposed siblings. Models were adjusted for the above-mentioned covariates, stratified by family identification number, and used a robust sandwich estimator of SEs.^43^

Third, we assessed the extent to which lifetime psychiatric comorbidities could explain the association between PTSD and each educational milestone. To this end, the main analyses were repeated with a stepwise restriction in which we excluded individuals with comorbid psychiatric disorders (1 group at a time). In an additional ultrastringent analysis, models were further adjusted for all psychiatric comorbidities at the same time.

Fourth, we adjusted for general cognitive ability in the subset of men within each subcohort who underwent the conscription examination. Data management and analyses were performed using SAS, version 9.4 (SAS Institute Inc) and Stata, version 15.1 (StataCorp LLC), respectively. All tests used 2-tailed statistical significance set at *P* < .05.

## Results

### Descriptive Statistics

The final study cohort was composed of 2 244 193 individuals (1 151 414 men [51.3%] and 1 092 779 women [48.7%]). Descriptive characteristics of the study cohorts are listed in eTable 2 in the Supplement. In total, 919 of 1 425 326 individuals (0.1%) received a diagnosis of PTSD before being eligible to access upper secondary education, 2013 of 2 001 944 individuals (0.1%) received a diagnosis of PTSD before finishing upper secondary education, 2243 of 1 796 407 individuals (0.1%) received a diagnosis of PTSD before starting a university degree, and 2254 of 1 356 741 individuals (0.2%) received a diagnosis of PTSD before finishing a university degree. Across all 4 subcohorts, the proportion of women in the PTSD cohort (range, 77.2%-81.8%) was statistically significantly larger than among the cohort of individuals without PTSD (range, 48.3%-48.8%) (*P* < .001 for all comparisons). Individuals with PTSD presented more frequently with other psychiatric comorbidity compared with those without PTSD (range, 83.4%-85.1% vs 13.3%-14.0%, respectively) (*P* < .001 for all comparisons). Among conscripted men in each of the subcohorts, general cognitive ability was statistically significantly lower for those with a diagnosis of PTSD (range, 22-237 men) compared with those without PTSD (mean [SD], 3.9 [1.8] vs 5.1 [1.9] points, respectively) (*P* < .001 for all comparisons).

### Educational Milestones

Individuals with PTSD were statistically significantly less likely to complete each of the assessed educational milestones during the study period compared with individuals without PTSD. Regarding compulsory education, individuals diagnosed as having PTSD before the age of graduation (age range, 6-16 years) had 82% lower odds of being eligible to access upper secondary education compared with the individuals without a PTSD diagnosis (65.3% vs 91.3%, respectively; adjusted OR [aOR], 0.18; 95% CI, 0.15-0.20) (Table 1). For post–compulsory education, individuals who were diagnosed as having PTSD between ages 6 to 19 years had 87% lower odds of finishing upper secondary education (33.3% vs 80.5%, respectively; aOR, 0.13; 95% CI, 0.12-0.14) compared with those not diagnosed as having PTSD in this age interval. Similarly, individuals with a PTSD diagnosis recorded between ages 6 to 21 years had 68% lower odds of starting a university degree (15.9% vs 38.4%, respectively; aOR, 0.32; 95% CI, 0.28-0.35) compared with unexposed individuals. Those diagnosed as having PTSD between ages 6 to 25 years had 73% lower odds of finishing a university degree (8.6% vs 25.9%, respectively; aOR, 0.27; 95% CI, 0.23-0.31) compared with their unexposed counterparts. No sex differences were identified for any educational outcomes. When adjusting for completing the previous educational level, the estimates for each post–compulsory education outcome were attenuated but remained statistically significant (eTable 3 in the Supplement).

**Table 1.** Educational Attainment Among Individuals With PTSD Recorded Before the Corresponding Educational Milestone Compared With Unaffected Individuals From the General Population, Stratified by Sex

Variable	No. (%)^a^		OR (95% CI)^b^	
	Individuals with PTSD	Individuals without PTSD	Unadjusted model	Adjusted model^c^
Compulsory education				
Eligibility to access upper secondary education, No.	919	1 424 407	NA	NA
All	600 (65.3)	1 300 034 (91.3)	0.18 (0.16-0.21)	0.18 (0.15-0.20)
Women	468 (66.0)	642 738 (92.5)	0.16 (0.13-0.18)	0.17 (0.15-0.20)
Men	132 (62.9)	657 296 (90.1)	0.19 (0.14-0.25)	0.20 (0.15-0.26)
Post–compulsory education				
Finishing upper secondary education, No.	2013	1 999 931	NA	NA
All	670 (33.3)	1 610 765 (80.5)	0.12 (0.11-0.13)	0.13 (0.12-0.14)
Women	564 (34.3)	805 517 (82.8)	0.11 (0.10-0.12)	0.13 (0.11-0.14)
Men	106 (28.8)	805 248 (78.4)	0.11 (0.09-0.14)	0.14 (0.11-0.17)
Starting a university degree, No.	2243	1 794 164	NA	NA
All	357 (15.9)	688 378 (38.4)	0.30 (0.27-0.34)	0.32 (0.28-0.35)
Women	315 (17.2)	396 771 (45.7)	0.25 (0.22-0.28)	0.31 (0.28-0.35)
Men	42 (10.3)	291 607 (31.5)	0.25 (0.18-0.34)	0.33 (0.24-0.45)
Finishing a university degree, No.	2254	1 354 487	NA	NA
All	193 (8.6)	351 049 (25.9)	0.27 (0.23-0.31)	0.27 (0.23-0.31)
Women	172 (9.6)	220 339 (33.7)	0.21 (0.18-0.24)	0.27 (0.23-0.31)
Men	21 (4.6)	130 710 (18.7)	0.21 (0.13-0.32)	0.27 (0.18-0.42)

Abbreviations: NA, not applicable; OR, odds ratio; PTSD, posttraumatic stress disorder.

^a^ The denominators for the percentages of women and men with PTSD and without PTSD are the total number of women or men exposed and unexposed, respectively, in a corresponding subcohort. For example, in the subcohort for the analysis of finishing compulsory education, of 919 individuals with PTSD, 709 were women and 210 were men. These numbers were used as denominators for calculating the sex-specific percentages for those who achieved this milestone among exposed individuals (ie, [468 ÷ 709] × 100% = 66.0% for women and [132 ÷ 210] × 100% = 62.9% for men). The total number of exposed and unexposed individuals by sex within each subcohort is reported in eTable 2 in the Supplement.

^b^ All statistically significant.

^c^ Adjusted for sex, year of birth, maternal age at birth, and paternal age at birth.

In the sibling comparison models, the estimates for all educational milestones were considerably attenuated (aOR range, 0.22-0.53) compared with those in the main analyses (ie, nonoverlapping 95% CIs). However, individuals with PTSD still had lower odds of achieving all educational outcomes compared with their unaffected siblings (Table 2).

**Table 2.** Educational Attainment Among Individuals With PTSD Recorded Before the Corresponding Educational Milestone Compared With Their Unaffected Full Siblings

Variable	No. (%)^a^		OR (95% CI)^b^	
	Full siblings with PTSD	Full siblings without PTSD	Unadjusted model	Adjusted model^c^
Compulsory education				
No.	512	717	NA	NA
Eligibility to access upper secondary education	334 (65.2)	579 (80.8)	0.38 (0.28-0.51)	0.40 (0.27-0.60)
Post–compulsory education				
No.	1264	1916	NA	NA
Finishing upper secondary education	424 (33.5)	1182 (61.7)	0.24 (0.20-0.29)	0.22 (0.17-0.27)
No.	1407	2083	NA	NA
Starting a university degree	250 (17.8)	516 (24.8)	0.58 (0.48-0.71)	0.53 (0.41-0.68)
No.	1306	1881	NA	NA
Finishing a university degree	136 (10.4)	301 (16.0)	0.52 (0.40-0.67)	0.48 (0.35-0.66)

Abbreviations: NA, not applicable; OR, odds ratio; PTSD, posttraumatic stress disorder.

^a^ The denominators for the percentages of siblings with PTSD and without PTSD are the total number of exposed and unexposed siblings, respectively, in a corresponding subcohort. For example, in the subcohort for the analysis of finishing compulsory education, of 512 siblings with PTSD, 334 achieved this milestone. Therefore, the percentage among exposed siblings is 65.2% ([334 ÷ 512] × 100%).

^b^ All statistically significant.

^c^ Adjusted for sex, year of birth, maternal age at birth, and paternal age at birth.

For each outcome, systematically excluding individuals with different groups of psychiatric comorbidity 1 at a time did not statistically significantly alter the results (aOR range, 0.13-0.38) (Table 3). In addition, an ultrastringent analysis in which we further adjusted for all psychiatric comorbidity at the same time resulted in attenuated but still statistically significant associations between PTSD and impaired educational outcomes except for the outcome of starting a university degree in the sibling comparison (aOR range, 0.41-0.76) (eTable 4 in the Supplement).

**Table 3.** Educational Attainment Among Individuals With PTSD Recorded Before the Corresponding Educational Milestone Compared With Unaffected Individuals From the General Population, Excluding Various Groups of Psychiatric Comorbidities

Variable	Adjusted OR (95% CI)^a^							
	Whole cohort	Disorders excluded						
		Neurodevelopmental	Conduct	Anxiety	Affective	Eating	Psychotic	Substance use
Compulsory education								
Eligibility to access upper secondary education	0.18 (0.15-0.20)	0.21 (0.18-0.26)	0.19 (0.16-0.22)	0.18 (0.14-0.22)	0.18 (0.14-0.23)	0.17 (0.15-0.20)	0.19 (0.16-0.21)	0.19 (0.16-0.22)
Post–compulsory education								
Finishing upper secondary education	0.13 (0.12-0.14)	0.15 (0.13-0.16)	0.13 (0.12-0.15)	0.15 (0.13-0.17)	0.16 (0.14-0.18)	0.13 (0.12-0.14)	0.13 (0.12-0.15)	0.16 (0.14-0.18)
Starting a university degree	0.32 (0.28-0.35)	0.38 (0.34-0.43)	0.32 (0.29-0.36)	0.38 (0.32-0.44)	0.30 (0.25-0.36)	0.30 (0.26-0.34)	0.32 (0.29-0.36)	0.37 (0.32-0.42)
Finishing a university degree	0.27 (0.23-0.31)	0.30 (0.26-0.35)	0.27 (0.23-0.31)	0.35 (0.28-0.43)	0.29 (0.23-0.36)	0.27 (0.23-0.32)	0.29 (0.25-0.33)	0.31 (0.27-0.37)

Abbreviations: OR, odds ratio; PTSD, posttraumatic stress disorder.

^a^ All statistically significant. The ORs (95% CIs) are adjusted for sex, year of birth, maternal age at birth, and paternal age at birth.

When restricting analyses to conscripted men, the estimates were similar to those observed for men in the corresponding main analyses (eTable 5 in the Supplement). When models were also adjusted for the level of general cognitive ability, the estimates were slightly attenuated but remained statistically significant for all educational milestones except for the outcome of starting a university degree, which was no longer statistically significant, likely because of insufficient power (aOR range, 0.19-0.68) (eTable 5 in the Supplement).

## Discussion

The main finding in this population-based cohort study is that individuals with PTSD were consistently less likely to achieve all of the educational milestones studied, spanning from compulsory education to finishing a university degree, compared with individuals from the general population. Although attenuated, the results remained statistically significant after strict control for important confounders, including shared familial factors, psychiatric comorbidity, and general cognitive ability.

In this study, a preceding PTSD diagnosis seemed to be most associated with not completing upper secondary education. The odds of achieving this milestone were 87% lower for individuals with PTSD compared with those without PTSD. In other words, only 33% of individuals with PTSD completed this level vs 81% of individuals without PTSD. The latter percentage is in line with that reported in 2019 by the Organisation for Economic Cooperation and Development,^44^ which indicated that 83% of adults in Sweden aged 25 to 64 years completed upper secondary education. Similarly, the results of the present study showed that individuals with PTSD had 68% lower odds of starting a university degree and 73% lower odds of finishing a university degree compared with individuals without the disorder within the same age range. These results match those of previous much smaller studies^18,19,45,46^ reporting that PTSD plays a role in whether students remain enrolled in university.

In the sibling comparison, the results remained statistically significant, but the magnitude of the ORs approximately halved. This attenuation suggests that shared familial factors are important in explaining the association between PTSD and educational attainment. Therefore, it is possible that shared genetic associations may partially explain both a higher risk of PTSD and diminished educational performance in the same individuals who present both.^15,47,48^ Environmental risk factors shared by siblings, such as socioeconomic status, parental psychopathology, or parental educational level (which have been previously associated with school performance in the offspring in their own right^49,50^), may be additional contributing factors.

Systematically removing various groups of psychiatric disorders from the analyses did not substantially alter the results. This finding is in contrast to a previous much smaller study^17^ that reported worse educational outcomes in individuals with self-reported PTSD and alcohol use compared with those with PTSD alone. An ultrastringent analysis with adjustment for all psychiatric comorbidities at the same time resulted in somewhat attenuated estimates, but the lower odds of finishing the milestones for individuals with PTSD still held. Therefore, strict adjustment for psychiatric comorbidities did not explain the associations observed in this study.

In line with previous literature suggesting that lower premorbid intelligence is a risk factor for PTSD,^2,26^ conscripted men diagnosed as having PTSD had statistically significantly lower general cognitive ability compared with those without PTSD. Therefore, adjusting for general cognitive ability was an important addition to the analyses in the present study. After adjusting for general cognitive ability, men with PTSD still had worse academic performance across the various milestones except for the association with starting a university degree, which was not statistically significant, probably because of limited power.

These results suggest that PTSD is associated with profound impairments in educational performance over and above familial factors, psychiatric comorbidity, and general cognitive ability. Although the results are not specific to PTSD—academic difficulties have also been described in other psychiatric disorders using similar methods^22,23,24,25^—the association of PTSD with educational performance seems to be more pronounced than in these other conditions, such as social anxiety disorder or obsessive-compulsive disorder.^23,25^ Presumably, the core symptoms of PTSD, such as reexperiencing, hyperarousal, dissociation, and sleep problems,^51^ as well as their downstream consequences on attentional or memory resources,^52^ substantially interfere with the ability to function academically.

The wider implications of the results in this study are worth considering. Raising awareness in schools about the consequences that trauma can have on students could motivate early referrals to mental health services; only one-half of those with severe PTSD receive treatment, and few receive specialist mental health care.^1^ Several evidence-supported training programs have been developed to integrate knowledge of trauma-related responses in teaching methods.^53,54^ These programs include, for example, the Cognitive Behavioral Intervention for Trauma in Schools,^55,56^ a 10-week group and individual therapy program for parents and teachers,^57^ the Enhancing Resiliency Among Students Experiencing Stress (ERASE-Stress) program that has been reported to lower PTSD symptoms and depression among students,^58^ and the RAP Club 12-session, school-based, trauma-informed group intervention based on cognitive behavior therapy and mindfulness strategies.^59^

### Strengths and Limitations

This study has multiple strengths. First is the inclusion of a large, population-based cohort with objective educational outcome data collected prospectively from nationwide administrative records of a universal educational system. Second, the diagnostic codes for PTSD in the NPR have high validity and reliability.^40^ Third, the sibling comparison design allowed us to control for unmeasured confounders shared by full siblings.^60^ Fourth, we were able to strictly control for the role of psychiatric comorbidity and general cognitive ability.

The study also has limitations. First, analyses are based on treatment-seeking individuals diagnosed by specialists, which may affect the generalizability of the findings. Individuals with PTSD tend to seek help late after onset of symptoms,^61,62^ which may imply a delay in diagnosis associated with misclassifications in exposed vs unexposed individuals. Furthermore, outpatient records were available only from 2001 onward. Second, the NPR does not include information on the type or number of traumatic events or any measures of symptom severity, which could potentially alter the magnitude of the observed educational impairment. Third, adjustment for general cognitive ability could be performed only in men because data for women in the Conscription Register are scarce. Whether the same results generalize to women remains to be explored. Fourth, sibling comparisons include some inherent limitations, such as potential carryover associations and environmental confounders varying between siblings.^60^

## Conclusions

This study found that posttraumatic stress disorder was associated with impaired educational performance across the life span independent of familial factors shared between siblings, psychiatric comorbidity, and general cognitive ability. This finding highlights the importance of implementing trauma-informed interventions in schools and universities to minimize the long-term socioeconomic consequences of academic failure.

## Notes

Supplement. **eTable 1.** Diagnostic Groups With International Classification of Diseases, Version 8 (ICD-8), 9 (ICD-9), and 10 (ICD-10) Codes and Age Thresholds for Psychiatric Disorders and Excluded Diagnoses **eTable 2.** Distribution of Study Covariates Among Individuals With Post-traumatic Stress Disorder (PTSD) and Unaffected Individuals From the General Population Within Each Subcohort **eTable 3.** Odds Ratios (OR) and Corresponding 95% CIs for Educational Attainment Among Individuals With Post-traumatic Stress Disorder (PTSD) Recorded Before the Corresponding Educational Milestone, Compared With Unaffected Individuals From the General Population, Stratified by Gender and Controlled for the Achievement at Previous Educational Level **eTable 4.** Odds Ratios (OR) and Corresponding 95% CIs for Educational Attainment Among Individuals With Post-traumatic Stress Disorder (PTSD) Recorded Before the Corresponding Educational Milestone, Compared With Unaffected Individuals From the General Population (Stratified by Gender) and Compared With Their Unaffected Full Siblings, and Adjusted for All Psychiatric Disorders at the Same Time **eTable 5.** Odds Ratios (OR) and Corresponding 95% CIs for Educational Attainment Among Males With Cognitive Ability Measures Available From Conscription Examination With Post-traumatic Stress Disorder (PTSD) Recorded Before the Corresponding Educational Milestone, Compared With Unaffected Individuals From the General Population
