# Supplementary material for: Assessment of Posttraumatic Stress Disorder and Educational Achievement in Sweden
Source: JAMA Netw Open. 2020 Dec 8;3(12):e2028477. doi: 10.1001/jamanetworkopen.2020.28477 (PMC7724559; doi:10.1001/jamanetworkopen.2020.28477)
Supplement: Supplement. — eTable 1. Diagnostic Groups With International Classification of Diseases, Version 8 (ICD-8), 9 (ICD-9), and 10 (ICD-10) Codes and Age Thresholds for Psychiatric Disorders and Excluded Diagnoses eTable 2. Distribution of Study Covariates Among Individuals With Post-traumatic Stress Disorder (PTSD) and Unaffected Individuals From the General Population Within Each Subcohort eTable 3. Odds Ratios (OR) and Corresponding 95% CIs for Educational Attainment Among Individuals With Post-traumatic Stress Disorder (PTSD) Recorded Before the Corresponding Educational Milestone, Compared With Unaffected Individuals From the General Population, Stratified by Gender and Controlled for the Achievement at Previous Educational Level eTable 4. Odds Ratios (OR) and Corresponding 95% CIs for Educational Attainment Among Individuals With Post-traumatic Stress Disorder (PTSD) Recorded Before the Corresponding Educational Milestone, Compared With Unaffected Individuals From the General Population (Stratified by Gender) and Compared With Their Unaffected Full Siblings, and Adjusted for All Psychiatric Disorders at the Same Time eTable 5. Odds Ratios (OR) and Corresponding 95% CIs for Educational Attainment Among Males With Cognitive Ability Measures Available From Conscription Examination With Post-traumatic Stress Disorder (PTSD) Recorded Before the Corresponding Educational Milestone, Compared With Unaffected Individuals From the General Population [file jamanetwopen-e2028477-s001.pdf]

Supplementary Online Content

Vilaplana-Pérez A, Sidorchuk A, Pérez-Vigil A, et al. Assessment of posttraumatic stress disorder and educational achievement in Sweden. *JAMA Netw Open*. 2020;3(12):e2028477. doi:10.1001/jamanetworkopen.2020.28477

eTable 1. Diagnostic Groups With International Classification of Diseases, Version 8 (ICD-8), 9 (ICD-9), and 10 (ICD-10) Codes and Age Thresholds for Psychiatric Disorders and Excluded Diagnoses

eTable 2. Distribution of Study Covariates Among Individuals With Post-traumatic Stress Disorder (PTSD) and Unaffected Individuals From the General Population Within Each Subcohort

eTable 3. Odds Ratios (OR) and Corresponding 95% CIs for Educational Attainment Among Individuals With Post-traumatic Stress Disorder (PTSD) Recorded Before the Corresponding Educational Milestone, Compared With Unaffected Individuals From the General Population, Stratified by Gender and Controlled for the Achievement at Previous Educational Level

eTable 4. Odds Ratios (OR) and Corresponding 95% CIs for Educational Attainment Among Individuals With Post-traumatic Stress Disorder (PTSD) Recorded Before the Corresponding Educational Milestone, Compared With Unaffected Individuals From the General Population (Stratified by Gender) and Compared With Their Unaffected Full Siblings, and Adjusted for All Psychiatric Disorders at the Same Time

eTable 5. Odds Ratios (OR) and Corresponding 95% CIs for Educational Attainment Among Males With Cognitive Ability Measures Available From Conscription Examination With Post-traumatic Stress Disorder (PTSD) Recorded Before the Corresponding Educational Milestone, Compared With Unaffected Individuals From the General Population

This supplementary material has been provided by the authors to give readers additional information about their work.

© 2020 Vilaplana-Pérez A et al. *JAMA Network Open*.

eTable 1. Diagnostic groups with International Classification of Diseases, version 8 (ICD-8), 9 (ICD-9), and 10 (ICD-10) codes and age thresholds for psychiatric disorders and excluded diagnoses.

**Psychiatric disorders**	**ICD codes**	**Minimal age of diagnosis**
**Neurodevelopmental disorders**, including autism spectrum disorders, attention-deficit/hyperactivity disorder[a], Tourette syndrome and chronic tic disorder, and learning disabilities	ICD-8 306.2<br>ICD-9 299, 307C, 314<br>ICD-10 F81, F84, F90, F95<br>ADHD drugs:[a] N06BA01, N06BA02, N06BA04, N06BA09	≥ 3 years old<br>(≥1 year old for autism spectrum disorders)
**Conduct disorder**	ICD-8 and ICD-9 n/a<br>ICD-10 F91	≥3 years old
**Phobic, anxiety, and obsessive-compulsive disorders**	ICD-8 300.00, 300.20, 300.3<br>ICD-9 300A, 300C, 300D<br>ICD-10 F40, F41, F42	≥6 years old
**Affective disorders**, including bipolar, depressive, and persistent mood disorders	ICD-8 296, 298.09<br>ICD-9 296, 298A, 300E, 311<br>F25.0, F30-F39	≥6 years old<br>(≥10 year old for bipolar disorder)
**Eating disorders**	ICD-8 n/a<br>ICD-9 307B, 307F<br>ICD-10 F50.0-F50.3, F50.9	≥8 years old
**Psychotic disorders**, including schizophrenia, schizotypal, and delusional disorders	ICD-8 and ICD-9 295 (minus 295.5/ 295F), 297, 298 (minus 298.09/298A)<br>ICD-10 F20, F21, F22, F23, F24, F25 (minus F25.0), F28, F29	≥10 years old
**Substance use disorders**	ICD-8 303, 304<br>ICD-9 303, 304, 305A, 305X<br>ICD-10 F10-F16 and F18-19 (minus F1x.5)	≥10 years old
**Exclusion groups**	**ICD codes**	**Minimal age of diagnosis**
**Organic brain disorder**	ICD-8 and ICD-9 290-294 (minus 294.3)<br>ICD-10 F00-F09	≥1 year old
**Intellectual disabilities**	ICD-8 311-315<br>ICD-9 317-319<br>ICD-10 F70-F79	≥1 year old

[a]Individuals with attention-deficit/hyperactivity disorder (ADHD) were also identified by prescription of ADHD drugs, collected from the Prescription Drug Register, specifically Amfetamine (Anatomical Therapeutic Chemical [ATC] Classification System code: N06BA01), Dexamfetamine (N06BA02), Methylphenidate (N06BA04), and Atomoxetine (N06BA09).

© 2020 Vilaplana-Pérez A et al. *JAMA Network Open*.

eTable 2. Distribution of study covariates among individuals with post-traumatic stress disorder (PTSD) and unaffected individuals from the general population within each subcohort.

Covariates[a]	Eligibility to access upper secondary education		Finishing upper secondary education		Starting a university degree		Finishing a university degree	
	Individuals with PTSD n=919	Individuals without PTSD n=1,424,407	Individuals with PTSD n=2,013	Individuals without PTSD n=1,999,931	Individuals with PTSD n=2,243	Individuals without PTSD n=1,794,164	Individuals with PTSD n=2,254	Individuals without PTSD n=1,354,487
	n (%)	n (%)	n (%)	n (%)	n (%)	n (%)	n (%)	n (%)
Women	709 (77.2)	694,763 (48.8)	1,645 (81.7)	972,687 (48.6)	1,834 (81.8)	869,154 (48.4)	1,792 (80)	653,962 (48.3)
Age of mothers at birth of index person, mean (SD), years	27.3 (5.4)	28.2 (5.0)	27.3 (5.5)	27.6 (5.0)	26.9 (5.5)	27.5 (5.0)	27.0 (5.5)	27.2 (5.0)
Missing, n (%)	0	0	0	0	0	0	0	0
Age of fathers at birth of index person, mean (SD), years	30.4 (6.6)	31 (5.9)	30.4 (6.7)	30.3 (5.8)	30.1 (6.6)	30.2 (5.8)	30.2 (6.5)	30.0 (5.7)
Missing, n (%)	6 (0.6)	6,056 (0.4)	11 (0.6)	10,020 (0.5)	16 (0.7)	9,066 (0.5)	22 (0)	7,080 (0.5)
Any comorbid disorder	766 (83.4)	198,945 (14.0)	1,691 (84.0)	272,219 (13.6)	1,888 (84.2)	244,178 (13.6)	1,919 (85.1)	179,627 (13.3)
Neurodevelopmental disorders	304 (33.1)	58,634 (4.1)	552 (27.4)	62,416 (3.1)	572 (25.5)	51,132 (2.9)	501 (22.2)	32,119 (2.4)
Conduct disorders	72 (7.8)	4,128 (0.3)	88 (4.4)	3,458 (0.2)	68 (3.1)	2,507 (0.1)	29 (1.3)	1,120 (0.1)
Anxiety disorders and obsessive-compulsive disorder	438 (47.7)	78,380 (5.5)	1,117 (55.5)	115,860 (5.8)	1,296 (57.8)	106,374 (5.9)	1,386 (61.5)	81,334 (6.0)
Affective disorders	554 (60.3)	78,054 (5.5)	1,252 (62.2)	119,932 (6.0)	1,392 (62.1)	110,485 (6.2)	1,415 (62.8)	85,883 (6.3)
Eating disorders	93 (10.1)	18,089 (1.3)	260 (12.9)	21,393 (1.1)	296 (13.2)	18,704 (1.0)	317 (14.1)	12,651 (0.9)
Schizophrenia, schizotypal, delusional	29 (3.2)	5,799 (0.4)	145 (7.2)	11,716 (0.6)	183 (8.2)	11,227 (0.6)	207 (9.2)	9,335 (0.7)
Substance use disorders	202 (22.0)	64,522 (4.5)	636 (31.6)	92,976 (4.7)	752 (33.5)	85,030 (4.7)	785 (34.8)	62,201 (4.6)
Men conscripted with general cognitive ability measure, n (%)	22 (2.4)	271,266 (19.0)	79 (3.9)	645,528 (32.3)	120 (5.4)	642,126 (35.8)	237 (10.5)	575,865 (42.5)
General cognitive ability at examination, mean (SD)	3.9 (1.8)	5.1 (1.9)	3.9 (1.8)	5.1 (1.9)	3.9 (1.8)	5.1 (1.9)	3.9 (1.8)	5.1 (1.9)

[a] For all covariates, a statistically significant between-group difference ($p<0.001$) was determined with a χ^2 test and an independent-sample 2-tailed t-test for categorical and continuous variables, respectively.

© 2020 Vilaplana-Pérez A et al. *JAMA Network Open*.

eTable 3. Odds ratios (OR) and corresponding 95% confidence intervals (CI) for educational attainment among individuals with post-traumatic stress disorder (PTSD) recorded before the corresponding educational milestone, compared with unaffected individuals from the general population, stratified by gender and controlled for the achievement at previous educational level.

	**Individuals with PTSD**	**Individuals without PTSD**	**Unadjusted model**	**Adjusted model[a]**	**Additionally adjusted for previous educational level[b]**
	**n (%)**	**n (%)**	**OR (95% CI)**	**OR (95% CI)**	**OR (95% CI)**
**Post-compulsory education**					
**Finishing upper secondary education**	**n=1,770**	**n= 1,191,111**			
All	611 (34.5)	942,519 (79.1)	**0.14 (0.13-0.15)**	**0.12 (0.11-0.14)**	**0.14 (0.13-0.16)**
Women	516 (35.4)	473,930 (81.7)	**0.12 (0.11-0.14)**	**0.12 (0.11-0.14)**	**0.14 (0.12-0.16)**
Men	95 (30.3)	468,589 (76.7)	**0.13 (0.10-0.17)**	**0.13 (0.10-0.17)**	**0.15 (0.11-0.19)**
**Starting a university degree**	**n=2,243**	**n=1,794,164**			
All	357 (15.9)	688,378 (38.4)	**0.30 (0.27-0.34)**	**0.32 (0.28-0.35)**	**0.56 (0.50-0.64)**
Women	315 (17.2)	396,771 (45.7)	**0.25 (0.22-0.28)**	**0.31 (0.28-0.35)**	**0.56 (0.49-0.63)**
Men	42 (10.3)	291,607 (31.5)	**0.25 (0.18-0.34)**	**0.33 (0.24-0.45)**	**0.59 (0.43-0.82)**
**Finishing a university degree**	**n=2,254**	**n=1,354,487**			
All	193 (8.6)	351,049 (25.9)	**0.27 (0.23-0.31)**	**0.27 (0.23-0.31)**	**0.41 (0.35-0.49)**
Women	172 (9.6)	220,339 (33.7)	**0.21 (0.18-0.24)**	**0.27 (0.23-0.31)**	**0.41 (0.34-0.49)**
Men	21 (4.6)	130,710 (18.7)	**0.21 (0.12-0.32)**	**0.27 (0.18-0.42)**	**0.49 (0.31-0.79)**

[a] Adjusted for sex, year of birth, maternal age at birth, and paternal age at birth.
[b] Estimates are additionally adjusted for achievement at previous educational level such as: estimates for finishing upper secondary education are adjusted for eligibility to access upper secondary education; estimated for starting a university degree are adjusted for finishing upper secondary education; and estimates for finishing a university degree are adjusted for starting a university degree.
Note: Analyses for each specific educational outcome are performed in corresponding subgroups among individuals with information on achievement at previous educational level. Statistically significant findings are highlighted in bold.
Abbreviations: CI confidence interval; OR odds ratio; PTSD post-traumatic stress disorder

© 2020 Vilaplana-Pérez A et al. *JAMA Network Open*.

eTable 4. Odds ratios (OR) and corresponding 95% confidence intervals (CI) for educational attainment among individuals with post-traumatic stress disorder (PTSD) recorded before the corresponding educational milestone, compared with unaffected individuals from the general population (stratified by gender) and compared with their unaffected full siblings, and adjusted for all psychiatric disorders at the same time.

	Comparison with general population			Sibling comparison	
	**Unadjusted model**	**Adjusted model[a]**	**Additionally adjusted for "all comorbidities"[b]**	**Unadjusted model[a]**	**Additionally adjusted for "all comorbidities"[b]**
	**OR (95% CI)**	**OR (95% CI)**	**OR (95% CI)**		
**Compulsory education**					
**Eligibility to access upper secondary education**					
All	**0.18 (0.16-0.21)**	**0.18 (0.15-0.20)**	**0.38 (0.33-0.44)**	**0.40 (0.27-0.60)**	**0.57 (0.37-0.86)**
Women	**0.16 (0.13-0.18)**	**0.17 (0.15-0.20)**	**0.37 (0.31-0.44)**	–	–
Men	**0.19 (0.14-0.25)**	**0.20 (0.15-0.26)**	**0.43 (0.32-0.58)**	–	–
**Post-compulsory education**					
**Finishing upper secondary education**					
All	**0.12 (0.11-0.13)**	**0.13 (0.12-0.14)**	**0.30 (0.27-0.33)**	**0.22 (0.17-0.27)**	**0.41 (0.32-0.53)**
Women	**0.11 (0.10-0.12)**	**0.13 (0.11-0.14)**	**0.30 (0.27-0.33)**	–	–
Men	**0.11 (0.09-0.14)**	**0.14 (0.11-0.17)**	**0.29 (0.23-0.37)**	–	–
**Starting a university degree**					
All	**0.30 (0.27-0.34)**	**0.32 (0.28-0.35)**	**0.47 (0.42-0.53)**	**0.53 (0.41-0.68)**	0.76 (0.55-1.05)
Women	**0.25 (0.22-0.28)**	**0.31 (0.28-0.35)**	**0.45 (0.40-0.50)**	–	–
Men	**0.25 (0.18-0.34)**	**0.33 (0.24-0.45)**	**0.51 (0.37-0.70)**	–	–
**Finishing a university degree**					
All	**0.27 (0.23-0.31)**	**0.27 (0.23-0.31)**	**0.44 (0.38-0.51)**	**0.48 (0.35-0.66)**	**0.51 (0.34-0.78)**
Women	**0.21 (0.18-0.24)**	**0.27 (0.23-0.31)**	**0.42 (0.36-0.49)**	–	–
Men	**0.21 (0.13-0.32)**	**0.27 (0.18-0.42)**	**0.48 (0.31-0.74)**	–	–

[a] Adjusted for sex, year of birth, maternal age at birth, and paternal age at birth.
[b] Adjusted for sex, year of birth, maternal age at birth, and paternal age at birth and additionally adjusted for all comorbidities (including neuropsychiatric disorders, conduct disorders, anxiety disorders, affective disorders, eating disorders, psychotic disorders, and substance use disorders).
Note: Statistically significant findings are highlighted in bold.
Abbreviations: CI confidence interval; OR odds ratio.

© 2020 Vilaplana-Pérez A et al. *JAMA Network Open*.

eTable 5. Odds ratios (OR) and corresponding 95% confidence intervals (CI) for educational attainment among males with cognitive ability measures available from conscription examination with post-traumatic stress disorder (PTSD) recorded before the corresponding educational milestone, compared with unaffected individuals from the general population.

	**Individuals with PTSD**	**Individuals without PTSD**	**Unadjusted model**	**Adjusted model[a]**	**Additionally adjusted for cognitive ability measured at conscription**
	**n (%)**	**n (%)**	**OR (95% CI)**	**OR (95% CI)**	**OR (95% CI)**
**Compulsory education**					
Eligibility to access upper secondary education	**n=22**	**n=271,266**			
	16 (72.7)	254,129 (93.7)	**0.18 (0.07-0.46)**	**0.17 (0.06-0.44)**	**0.24 (0.06-0.87)**
**Post-compulsory education**					
Finishing upper secondary education	**n=79**	**n=645,528**			
	32 (40.5)	534,925 (82.9)	**0.14 (0.09-0.22)**	**0.15 (0.10-0.24)**	**0.19 (0.12-0.31)**
Starting a university degree	**n=120**	**n=642,126**			
	19 (15.8)	227,385 (35.4)	**0.34 (0.21-0.56)**	**0.40 (0.25-0.66)**	0.68 (0.41-1.12)
Finishing a university degree	**n=237**	**n=575,865**			
	15 (6.3)	115,551 (20.1)	**0.27 (0.16-0.45)**	**0.34 (0.20-0.58)**	**0.56 (0.32-0.96)**

[a] Adjusted for year of birth, maternal age at birth, and paternal age at birth.
Note: Statistically significant findings are highlighted in bold.
Abbreviations: CI confidence interval; OR odds ratio; PTSD post-traumatic stress disorder.

© 2020 Vilaplana-Pérez A et al. *JAMA Network Open*.
